# AMP5A modulates Toll-like receptors 7 and 8 single-stranded RNA immune responses in PMA-differentiated THP-1 and PBMC

**DOI:** 10.1186/s41231-022-00110-y

**Published:** 2022-03-03

**Authors:** Gregory Thomas, Kristen Hirter, Elizabeth Frederick, Melissa Hausburg, Raphael Bar-Or, Yetti Mulugeta, Michael Roshon, Charles Mains, David Bar-Or

**Affiliations:** 1 Ampio Pharmaceuticals Inc, 373 Inverness Parkway Suite 200, Englewood, CO 80122 USA; 2 Trauma Research Department, Swedish Medical Center, 501 E. Hampden, Englewood, CO 80113 USA; 3 Trauma Research Department, St. Anthony Hospital, 11600 W 2nd Pl, Lakewood, CO 80228 USA; 4 Trauma Research Department, Penrose Hospital, 2222 N Nevada Ave, Colorado Springs, CO 80907 USA; 5 Department of Molecular Biology, Rocky Vista University, 8401 S Chambers Rd, Parker, CO 80134 USA; 6 Centura Health Systems, 9100 E. Mineral Cir, Centennial, CO 80112 USA

**Keywords:** AMP5A, COVID-19, Autoimmunity, Toll-like receptor 7, Toll-like receptor 8, PPARγ, AhR, NF-κB, Cytokines, And Chemokines

## Abstract

**Background:**

Dysregulation of antiviral immunity has been implicated in the progression of acute respiratory syndrome coronavirus 2 infection into severe cases of coronavirus disease of 2019 (COVID-19). Imbalances in the inflammatory response drive the overabundant production of pro-inflammatory cytokines and chemokines. The low molecular weight fraction of 5% human serum albumin commercial preparation (AMP5A) is a novel biologic drug currently under clinical investigation for the treatment of osteoarthritis and the hyperinflammatory response associated with COVID-19. This study aims to elucidate AMP5A effects following the activation of immune cells with agonists of Toll-like receptor (TLR) 7 and/or 8, which detect ssRNA viral sequences.

**Methods:**

CXCL10 ELISAs were used to evaluate the dynamics of myeloid cells activated with CL075 and CL307, agonists of TLR7/8 and TLR7, respectively. In addition, enrichment analysis of gene sets generated by ELISA arrays was utilized to gain insight into the biologic processes underlying the identified differentially expressed cytokine profiles. Finally, relative potency (REP) was employed to confirm the involvement of mechanisms of action paramount to AMP5A activity.

**Results:**

AMP5A inhibits the release of CXCL10 from both CL075- and CL307-activated PMA-differentiated THP-1 and peripheral blood mononuclear cells. Furthermore, AMP5A suppresses a distinct set of pro-inflammatory cytokines (including IL-1β, IL-6, IL-12, and CXCL10) associated with COVID-19 and pro-inflammatory NF-κB activation. REP experiments using antagonists specific for the immunomodulatory transcription factors, peroxisome proliferator-activated receptor γ, and aryl hydrocarbon receptor, also indicate that these pathways are involved in the ability of AMP5A to inhibit CXCL10 release.

**Conclusion:**

Due to the biphasic course of COVID-19, therapeutic approaches that augment antiviral immunity may be more beneficial early in infection, whereas later interventions should focus on inflammation suppression. In this study, we show that AMP5A inhibits TLR 7/8 signaling in myeloid cells, resulting in a decrease in inflammatory mediators associated with hyperinflammation and autoimmunity. Furthermore, data demonstrating that AMP5A activates immunomodulatory transcription factors found to be protective in lung disease is provided. These findings suggest that the modes and mechanisms of action of AMP5A are well suited to treat conditions involving dysregulated TLR 7/8 activation.

**Graphical Abstract:**

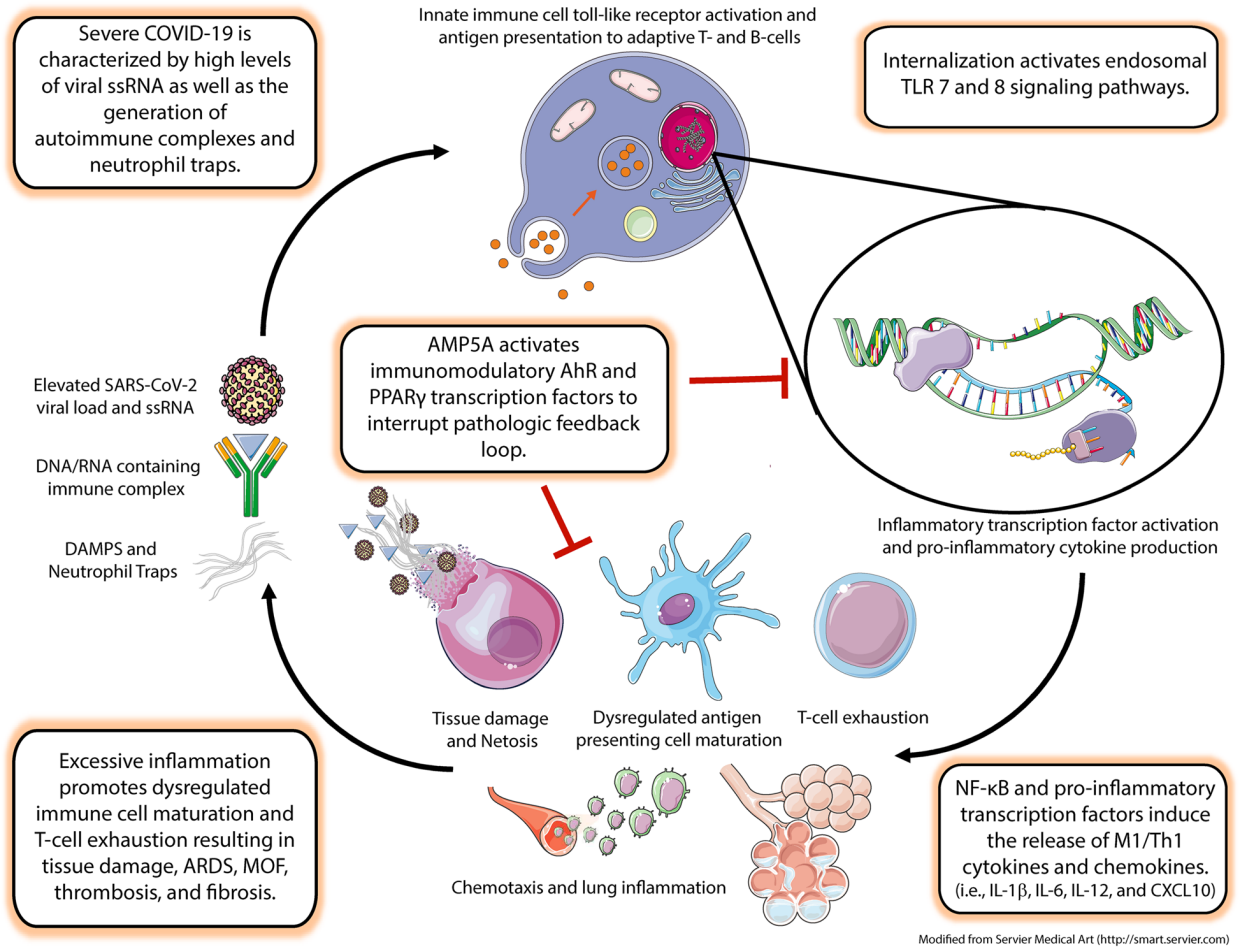

## Background

Mounting evidence suggests that clinical outcomes vary greatly for coronavirus disease of 2019 (COVID-19) depending on how the immune system responds to severe acute respiratory syndrome coronavirus 2 (SARS-CoV-2) infection. In its mildest forms, SARS-CoV-2 replication is primarily localized to the upper respiratory tract, with limited innate immune response and low viral burden [1]. However, underlying factors such as genetic polymorphisms, autoantibody development, and intrinsic viral mechanisms have been identified that serve to suppress or delay type I interferon production or activity during infection [2–4]. The resulting maladaptation in antiviral immunity appears to help drive progression into more severe stages marked by the migration of the virus to the lower respiratory tract, elevated viral loads, and dramatic loss of type II pneumocytes [1]. As the disease enters these phases, upregulation of pro-inflammatory mediators like CXCL10 become predictive of the clinical course and approximately 20% of these patients go on to develop acute respiratory distress syndrome (ARDS) [1]. Moreover, these critical stages are characterized by increased plasma levels of pro-inflammatory cytokines and chemokines, such as IFNγ, CXCL10, IL-1β, and TNFα, indicative of “cytokine storm” development that can eventually lead to multiorgan failure and death [5, 6]. Thus, it appears that failure of the immune system to mount an appropriate antiviral response early in infection drives a pathology involving excessive tissue damage, CD4+ T-cell helper type 1 (Th1) inflammation, and fibrosis.

Toll-like receptors (TLRs) are key pattern recognition complexes that help the immune system respond to pathogens based on unique microbial molecular structures [7]. However, tissue damage can result in the release of cellular components that mimic these molecular motifs and has been shown to play a role in the pathophysiology of diseases such as acute kidney injury through the activation of a wide range of TLRs [8, 9]. Furthermore, exuberant activation of TLRs following neutrophil extracellular trap (NETs) formation in mouse models is associated with acute lung injury [10] and has been postulated as a therapeutic target for the treatment of ARDS and cytokine storms in COVID- 19 [11]. Data also support the role of toll-like receptor (TLR) activation in autoimmune diseases such as rheumatoid arthritis and systemic lupus erythematosus (SLE) [12, 13]. Consequently, therapies that modulate and/or interrupt TLR signaling may prove beneficial for the treatment of diseases involving sterile inflammation and nucleic acid immune complex (IC) formation.

The low molecular weight fraction of 5% human serum albumin (AMP5A) is a novel anti-inflammatory biologic drug that has shown clinical efficacy for the treatment of osteoarthritis of the knee based on reductions in pain and improved overall function across multiple randomized, double-blinded human trials [14–16]. Influenced by these observations, clinical studies were designed to investigate the use of inhaled AMP5A for the treatment of the systemic inflammatory response syndrome and respiratory distress associated with COVID-19. Phase I trial results show that AMP5A treatment reduces all-cause COVID-19 mortality 5-fold (mortality of 24% in the standard of care group versus 5% in the AMP5A treatment group) [17].

*In vitro* investigations suggest that these clinical effects may result from the transcriptional modulation of inflammatory pathways. For instance, AMP5A demonstrates an ability to reduce the secretion of pro-inflammatory cytokines and chemokines associated with cytokine storms (i.e. IL-1β, IL-6, IL-12, TNFα, and CXCL10) from immune cells activated through TLRs and/or T-cells stimulated via the T-cell receptor [18–23]. Importantly, AMP5A inhibits nuclear factor kappa-light-chain-enhancer of activated B cells (NF-κB), based on reduced p65 and RelB subunit DNA binding activity, in lipopolysaccharide(LPS)-stimulated peripheral blood mononuclear cells (PBMC) [23]. Mechanistically, AMP5A has been shown to activate the immunomodulatory transcription factors aryl hydrocarbon receptor (AhR) and peroxisome proliferator activator receptor gamma (PPARγ) [19, 23] which convey antiviral activity and reduce lung inflammation in pulmonary viral infections [24, 25]. However, the immune system is inherently diverse, allowing for a myriad of functional configurations that can be tailored to a wide range of pathogens or insults, which can complicate the adoption of therapeutic strategies.

The purpose of this study was to confirm that myeloid cells, an immune subset important in mounting responses against viruses, express the molecular framework required for AMP5A activity. We also wanted to examine how AMP5A affects immune cells when they are activated via viral sensing mechanisms, as this could contribute to the hyperinflammation seen in severe COVID-19. In this study, we show that AMP5A inhibits TLR 7/8 signaling in myeloid cells, resulting in a decrease in inflammatory mediators associated with hyperinflammation and autoimmunity. Furthermore, data demonstrating that AMP5A activates immunomodulatory transcription factors found to be protective in lung disease is provided. These findings suggest that the modes and mechanisms of action of AMP5A are well suited to treat conditions involving dysregulated TLR 7/8 activation as well as improving clinical outcomes of COVID-19 by attenuating inflammation and reducing the potential for cytokine storm development and possibly fibrosis.

## Methods

### Materials

AMP5A was manufactured by Ampio Pharmaceuticals, Inc. (Englewood, CO). General cell culture reagents were purchased from ThermoFisher Scientific (Waltham, MA) while X-Vivo 15 serum-free medium was obtained from Lonza (Basel, Switzerland). 0.9% (w/v) sodium chloride was obtained from KD Medical (Columbia, MD). GW9662 and CH223191 antagonists were purchased from MilliporeSigma (St. Louis, MO) with 100 mM stock solutions prepared in DMSO and stored at -80°C prior to use. CL075 and CL307 were purchased from Invivogen (San Diego, CA) and stock solutions were prepared using supplied sterile, pyrogen-free water. ELISAs for IP10/CXCL10 (catalog # KAC2361) were purchased from ThermoScientific. All other reagents were obtained from MilliporeSigma (St. Louis, MO) unless otherwise stated.

### Cell culture and experimental treatments

Human THP-1 monocytes (ATCC, Manassas, VA) were passaged in RPMI 1640 media supplemented with 10% fetal bovine serum (FBS) and 1% penicillin/ streptomycin (Pen/ Strep) then differentiated using a final concentration of 50 ng/ml phorbol 12-myristate (PMA) in 96-well flat-bottom plates seeded at 1 × 10^5^ cells per well for 72 hours. Differentiation mediums were then aspirated and replenished as described below.

Both frozen and freshly isolated PBMC from consented donors were used in this investigation. When frozen stocks were utilized, cryopreserved vials obtained from Zen-Bio (Research Triangle Park, NC) were thawed using a Thawstar Automated Cell Thawing System (BioLife Solutions, Bothell, WA) and then transferred dropwise to RPMI 1640 medium containing 10% human AB serum, 1% penicillin-streptomycin (Pen/Strep), and 2 U/mL RNase-free DNase (ThermoScientific). Freshly isolated cells were isolated from sodium heparinized whole blood using Polymorphprep (Alere Technologies, Oslo, Norway) and washed with Dulbecco’s phosphate buffer saline. The resulting cell suspensions were then centrifuged at 1000 rpm for 10 minutes and working suspensions prepared at 2 x 10^6^ cells/mL in X-Vivo 15 or RPMI 1640 supplemented with 20% FBS, 2% Pen/Strep, 1% sodium bicarbonate, 7.5% solution, 1% 100 mM sodium pyruvate, 1% 100X MEM non-essential amino acid solution, and 1% 200 mM L-glutamine.

For experimental treatments, 100 µl of PBMC suspensions were added to 96-well tissue culture plates, or 100 µl of the 20% FBS RPMI medium described above was added to THP-1 plate wells. The solutions were then mixed with an equal volume of sterile 0.9% sodium chloride or AMP5A drug solutions and incubated at 37 °C and 5% CO_2_ for one hour. Stimulation was then achieved by adding CL075 or CL307 at final concentrations recommended by the manufacturer for an additional 24 to 72 hours before subsequent analysis. For relative potency investigation, 20 µM (10 µM final) concentration of antagonists were added to mediums prior to addition of saline or drug solutions serially diluted in saline.

### Cytokine and chemokine quantification

After the indicated stimulation periods, supernatants were collected for in-house CXCL10 ELISA measurements or sent to Eve Technologies (Calgary, Alberta, Canada) for human cytokine/chemokine 48-plex Discovery Assay Array analysis (catalog # HD48).

### Data and statistical analysis

Statistical analysis was performed using the Real Statistics Resource Pack Excel Add-in (http://www.real-statistics.com/) unless otherwise stated. For representative ELISAs, two-tailed, two-sample unequal variance student tests were used to compare groups in Microsoft Excel (Microsoft Corporation, Redmond, WA). For combined inhibition of release analysis, two-tailed, one-sample t-tests (hypothetical value = 0; α = 0.05) were used to establish meaningful percent inhibition measurements from vehicle controls. For cytokine arrays, two-tailed, one-sample t-tests were used to test for the significance of combined fold changes (hypothetical value = 1; α = 0.05). For potency assays, relative potency was calculated using 4P modeling with ANOVA pure separation, and similarity of dose responses was established by f-tests for non-parallelism, non-linearity, and significance of response in PLA 3.0 (Stegmann Systems GmbH, Raiffeisenstr, Germany). Box plots were generated, and descriptive statistics were performed, using BoxPlotR (shiny.chemgrid.org/boxplotr/). *In silico* pathway analysis of differentially expressed genes was performed using Enrichr (maayanlab.cloud/Enrichr) or Ingenuity Pathway Analysis (IPA) software (Qiagen Digital Insights, Redwood City, CA). For IPA, calculated differentially abundant cytokines/chemokines were uploaded, and a ‘Core’ expression analysis based on log-ratios for analytes with a p-value < 0.05 was run using our ‘user dataset’ as reference. IPA calculated overlap p-values and z-scores as confidence metrics when predicting canonical pathway associations. For overlap p-values, we considered the -log(p-value)>1.3 as significant. IPA calculated z-scores where z > 0 predicts activation, and z < 0 predicts inhibition; an absolute value of 2 was used as a significance cutoff.

## Results

### AMP5A reduces CL075- and CL307-induced CXCL10 release from PMA-differentiated THP-1 cells

**Fig. 1:**
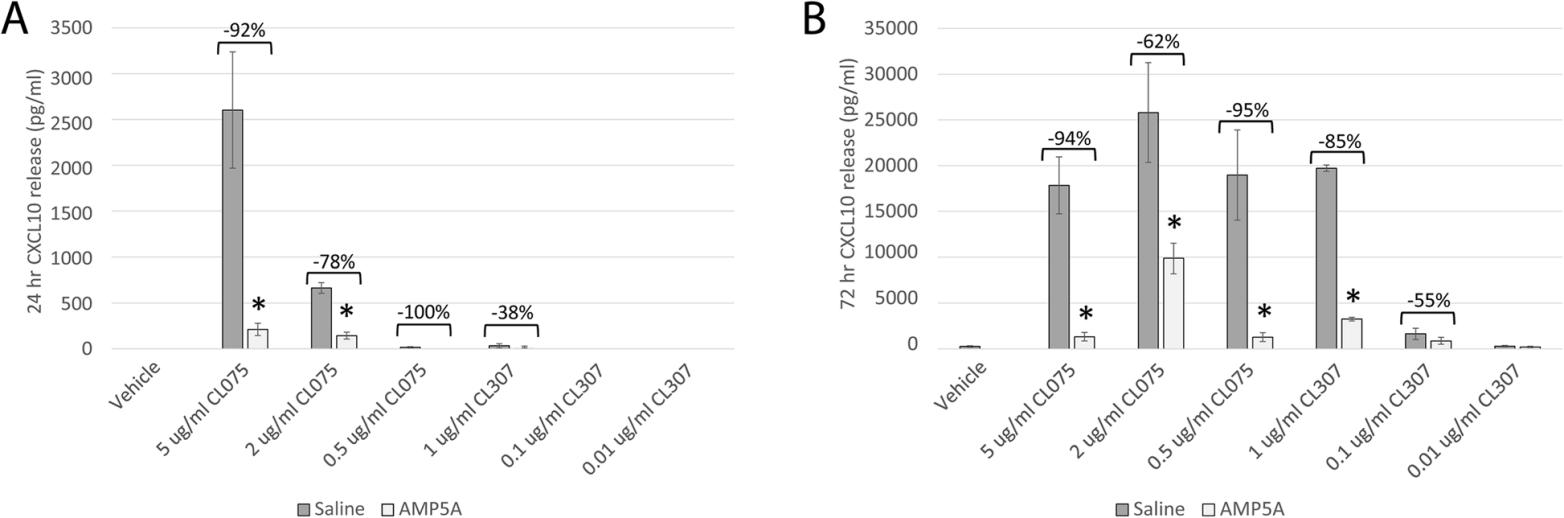
AMP5A inhibits CXCL10 release from THP-1 cells. Representative CL075- and CL307-induced CXCL10 chemokine release from PMA-differentiated THP-1 cells cultured in the presence of saline diluent control or AMP5A. Data presented as mean pg/ml CXCL10 ± STD of three technical replicates 24 hours (**A**) or 72 hours (**B**) post-stimulation. * = *p*-value ≤ 0.05 vs activated saline control by student t-test

One of the hallmarks of AMP5A anti-inflammatory activity is an ability to inhibit CXCL10 release from macrophage-like THP-1 monocytic cells following activation of TLR4 by LPS; accompanied by a reduction in the detectable amount of corresponding *CXCL10* messenger RNA [19]. To evaluate if this response extends to endosomal single-stranded ribonucleic acid (ssRNA) sensing pathways, CL075 and CL307 agonists were used to activate TLR7/8 or TLR7, respectively, in PMA-differentiated THP-1 cells with representative CXCL10 release results presented in Fig. 1.

When CXCL10 release was evaluated 24 hours post-stimulation with CL075, the dose-dependent release of chemokine was observed with significant (*p*-value ≤ 0.05) reductions of 92 ± 3% and 78 ± 6% by AMP5A treatment at final agonist concentrations of 5 µg/ml and 2 µg/ml, respectively (Fig. 1A). Complete attenuation of CXCL10 release was also observed with AMP5A treatment at 0.5 µg/ml levels of CL075 but this may have been the result of release measurements at or near the detection limit of the ELISA. As for CL307-induced CXCL10 from PMA-differentiated THP-1 cells, muted chemokine release was observed at this timepoint only at the highest concentration used (1 µg/ml) with a trend towards inhibition (38 ± 29%; *p*-value = 0.5). Dexamethasone (10 µM) and ibuprofen (100 µM) were also included as controls at this timepoint. While dexamethasone treatment resulted in similar inhibitions of CXCL10, cyclooxygenase targeting with ibuprofen showed no significant changes in chemokine release (data not shown).

Interestingly, different dose dynamics were observed when CXCL10 release was tested 72 hours post-stimulation (Fig. 1B). Indicative of an asymptotic dose-response, high levels of CL075-induced CXCL10 release were observed for all doses, with significant AMP5A inhibitions ranging from 62 ± 7% to 95 ± 3%. CXCL10 release in response to CL307 exposure at 72 hours was more vigorous and importantly, exhibited dose dependency. Drug-induced inhibitions of 84 ± 1% and 46 ± 23% were observed for 1 µg/ml and 0.1 µg/ml final concentrations of CL307, respectively, with the 1 µg/ml group exhibiting significance (p-value ≤ 0.05).

Together, these data provide evidence that AMP5A modulates CXCL10 release resulting from the activation of TLR7/8 in macrophage-like THP-1 cells.

### AMP5A reduces CL075- and CL307-induced CXCL10 release from PBMC

Similarly, AMP5A was also found to inhibit CXCL10 release from TLR4-activated PBMC [23]. To establish if AMP5A can influence *ex vivo* CL075- and CL307-induced CXCL10, these agonists were once again used to activate TLR7/8 in PBMC cocultures.

**Fig. 2:**
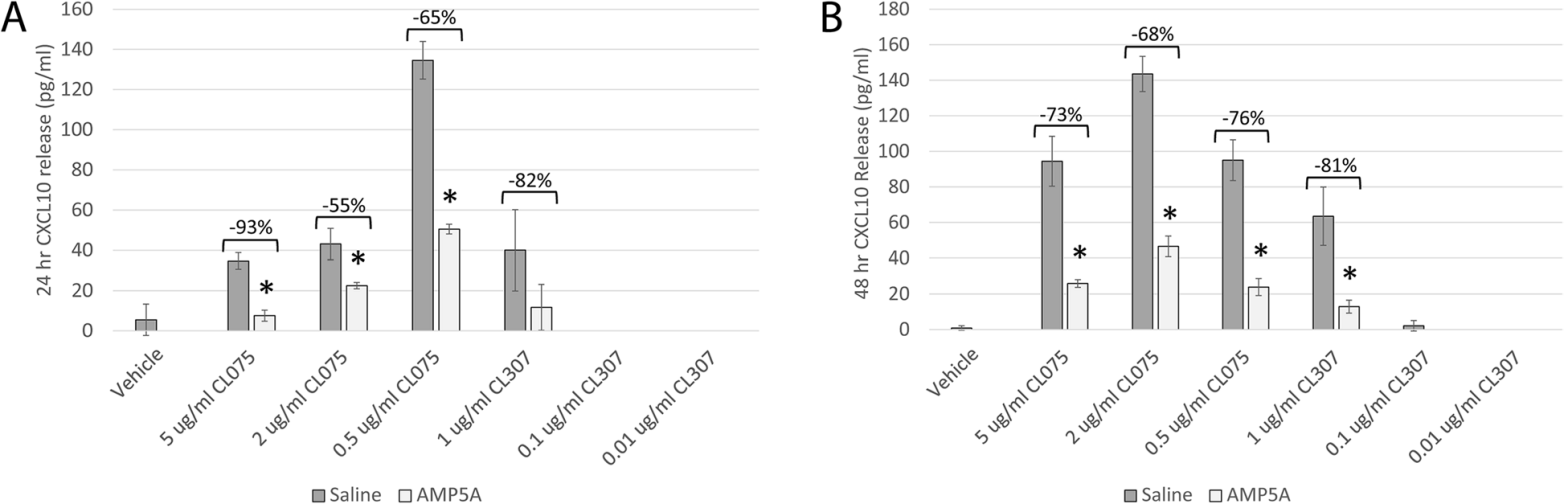
AMP5A inhibits CXCL10 release from PBMC. Representative CL075- and CL307-induced CXCL10 chemokine release from PBMC cultured in the presence of saline diluent control or AMP5A. Data presented as mean pg/ml CXCL10 ± STD of three technical replicates 24 hours (**A**) or 48 hours (**B**) post-stimulation. * = *p*-value ≤ 0.05 vs activated saline control by student t-test

As observed in PMA-differentiated THP-1, CXCL10 release induced by CL075 from PBMC is observed 24 hours following exposure (Fig. 2A). However, an inverse relationship of CXCL10 release to CL075 concentration was observed using the donor cells represented in Fig. 2A, suggesting a biphasic response. AMP5A treatment resulted in significant inhibitions of 93 ± 10%, 55 ± 4%, and 65 ± 2% in CXCL10 release from 5 µg/ml, 2 µg/ml, and 0.5 µg/ml concentrations of agonist, respectively when corrected for the background chemokine release observed for this donor. In contrast to THP-1, substantial CXCL10 release in response to a final concentration of 1.0 µg/ml CL307 was observed in PBMC cultures after 24 hours with a trend toward AMP5A-induced inhibition of 82 ± 33% (Fig. 2A). Measuring CXCL10 release from PBMC after 48 hours shows a similar asymptotic response compared to THP-1 cells with AMP5A inhibitions of 73 ± 2%, 68 ± 4%, and 76 ± 5% in CXCL10 release from 5 µg/ml, 2 µg/ml, and 0.5 µg/ml CL075, respectively (Fig. 2B). Significant inhibition of CXCL10 induced by 1.0 µg/ml CL307 is observed at this time point in the drug treatment group (81 ± 6%; p-value ≤ 0.05).

While the cells targeted in PBMC cultures still need to be characterized, these findings demonstrate AMP5A modulates TLR7/8 signaling in primary cells. In addition, we can infer that monocytic and/or other myeloid cells can also be targeted by AMP5A.

### Percent inhibition in CXCL10 release normalizes 24-hour CL075-induced PMA-differentiated THP-1 and PBMC data

**Fig. 3:**
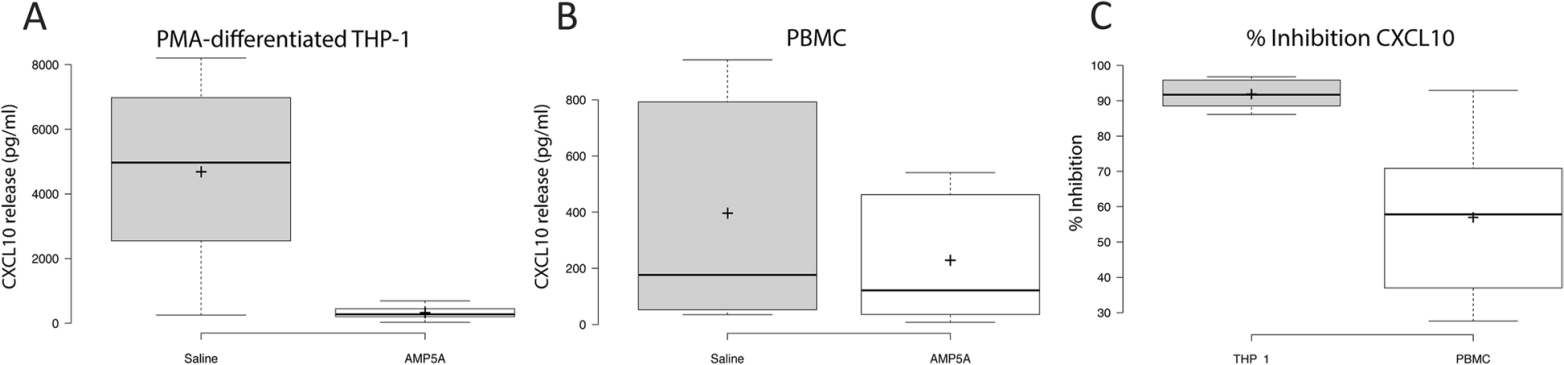
AMP5A % inhibition of CXCL10 release in THP-1 and PBMC. Box plots for 24-hour 5 µg/ml CL075-induced CXCL10 release and AMP5A percent inhibitions for PMA-differentiated THP-1 cells and PBMC. Data presented as CL075-induced CXCL10 pg/ml release for both saline- and AMP5A-treatment groups from PMA-differentiated THP-1 (**A**) or PBMC (**B**) as well as percent inhibitions in CXCL10 release observed in the AMP5A-treatment groups calculated versus saline-treated controls (**C**). Boxes = quartile 1 to 3 boundaries, line = Median, + = Mean. For THP-1, *n* = 8 independent experiments. For PBMC, *n* = 9 independent experiments using cells derived from a total of 7 different donors

Due to the magnitude and robustness of AMP5A inhibition in CXCL10 following 24-hour, 5 µg/ml CL075 activation, this treatment group was selected to determine variability and distributions in CXCL10 release. As shown in Fig. 3A, CL075-induced CXCL10 release from saline-treated PMA-differentiated THP-1 cells exhibits a wide, negatively skewed distribution (Median = 4967 pg/ml, IQR = 4432, *n* = 8) with a distinct reduction seen in the AMP5A treatment group (Median = 276 pg/ml, IQR = 244, *n* = 8). CL075-induced CXCL10 release from saline-treated PBMC also exhibits a wide distribution with a strong positive skew (Median = 177 pg/ml, IQR = 756, *n* = 9 across a total of 7 donors) (Fig. 3B). By comparison, the overall CXCL10 release appears lower in PBMC but this may reflect a lower number of targeted cells in these cultures as opposed to the THP-1 experiments. As expected, donor variability makes direct interpretation of raw release difficult but a reduction in CXCL10 release was observed (Median = 121 pg/ml, IQR = 427, *n* = 9).

Fig. 3C demonstrates how normalization within each experiment to percent inhibition in CXCL10 release versus control corrected for observed background chemokine release transforms the data to a more symmetrical distribution, making analysis more meaningful. Once drug response was calculated in this manner, AMP5A demonstrates an ability to inhibit CL075-induced CXCL10 release from PMA-differentiated THP-1 and PBMC by medians of 92% (IQR = 7, p-value ≤ 0.05 versus hypothetical 0) and 58% (IQR = 34, *p*-value ≤ 0.05 versus hypothetical 0) respectively.

Together, these data provide evidence that AMP5A modulates TLR7/8 signaling in both macrophage-like THP-1 cells and *ex vivo* PBMC cultures. Furthermore, the variability observed in overall CL075-induced CXCL10 release in PMA-differentiated THP-1 and PBMC models suggests that responses to TLR7/8 are highly dependent on cell-type and/or differentiation status. However, AMP5A inhibition of this pathway seems to be universal, regardless of these dynamics.

### PPARγ and AhR antagonists reduce AMP5A drug potency as measured by CL075-induced CXCL10 release

**Fig. 4:**
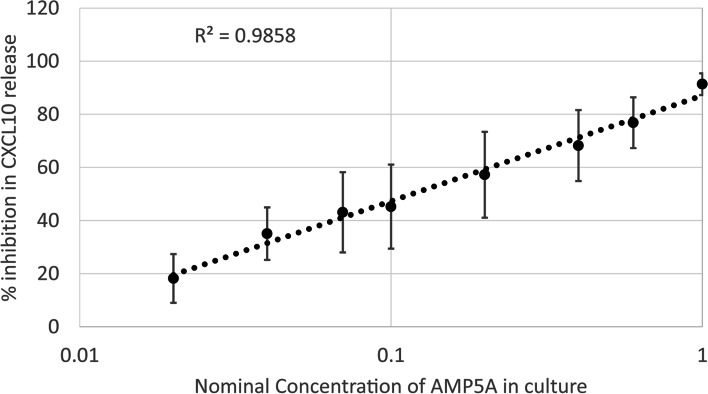
Relative potency assay of AMP5A inhibition of CXCL10. Dose-dependent reduction in CL075-induced CXCL10 release from PMA-differentiated THP-1 by AMP5A. Data presented as regression analysis of percent inhibitions ± STD in 24 hour, CL075-induced CXCL10 release versus AMP5A serially diluted using saline diluent with concentrations listed as nominal in relation to full strength drug product. *n* = 9 for nominal 1 or full-strength product and *n* = 4 for all other doses

Based on the dynamic range of the drug response described above, inhibition of CXCL10 release from PMA-differentiated THP-1 cells was chosen to investigate the involvement of pathways previously linked to the mechanism of actions of AMP5A. In the first step of this process, the drug product was serially diluted with saline vehicle and then reductions in 24-hour CL075-induced CXCL10 release were evaluated following percent inhibition normalization. Regression analysis demonstrates that AMP5A exhibits a log-linear, dose-dependent reduction (*R*^*2*^ = 0.986) in chemokine release conducive for relative potency (REP) calculation (Fig. 4). Next, the potency of AMP5A was established in the presence of antagonists for PPARγ (GW9662, MilliporeSigma) or AhR (CH223191, MilliporeSigma) as compared to DMSO vehicle controls. Exposure of cells to 10 µM final concentrations of GW9662 or CH223191 resulted in shifts in the log-linear dose-response of AMP5A-induced CXCL10 inhibition reflective of a loss in drug potency (data not shown). REP calculated for 3 independent experiments demonstrates that antagonism of GW9662 and CH223191 results in reduced mean drug potency to 0.29 ± 0.06 and 0.40 ± 0.18, respectively (Table 1). These findings infer that AMP5A activates PPARγ and AhR transcription factors to inhibit the release of CXCL10 from PMA-differentiated THP-1 cells stimulated with CL075.

**Table 1:** Disruption of PPARγ or AhR signaling decreases the relative potency of AMP5A. Effect of GW9662 or CH223191 antagonism on AMP5A drug potency. Data presented as mean relative potency (REP) ± STD and 95% CI of three independent experiments versus DMSO controls

Antagonist (Pathway)	Mean REP ± STD (95% Cl)	REP 1 (95% Cl)	REP 2 (95% Cl)	REP 3 (95% Cl)
GW9662 (PPARy)	0.29 ± 0.06 (0.22 – 0.36)	0.26 (0.19 – 0.37)	0.24 (0.09 – 0.66)	0.36 (0.16 – 0.83)
CH223191 (AhR)	0.40 ± 0.18 (0.21 – 0.60)	0.21 (0.13- 0.32)	0.56 (0.35 – 0.90)	0.43 (0.21 – 0.86)

### ELISA array analysis demonstrates that AMP5A inhibits the release of a diverse set of pro-inflammatory cytokines and chemokines from CL075-activated, PMA-differentiated THP-1 cells

To help qualify the overall immune response in PMA-differentiated THP-1 cells following CL075 activation and identify additional drug effects, a multi-plex ELISA array was employed to assess the release of 48 cytokines, chemokines, and growth factors. Using this expanded format, a total of 26 inflammatory mediators were found to be significantly (p ≤ 0.05) inhibited by AMP5A-treatment 24 hours following activation with 5 µg/ml CL075 (Table 2). Consistent with the previous findings in this investigation, CXCL10 proved to be the most highly downregulated (97 ± 2% inhibition) target on the array. Other prominent pro-inflammatory cytokines such as IL-6 (92 ± 9%), IL-12 (p40 = 91 ± 1%; p70 = 63 ± 7%), IL-1β (70 ± 10%), and TNF-α (56 ± 5%) as well as chemokines including MCP (MCP-1 = 90 ± 1%; MCP-3 = 86 ± 5%), MIP (MIP-1β = 70 ± 10%; MIP-1α = 68 ± 5%), CXCL9 (57 ± 11%) and RANTES (53 ± 1%) were also significantly suppressed. Of note, some reduction in type I and type II interferon was also observed but did not prove significant vs controls (data not shown).

**Table 2:** Multiple cytokines and chemokines are significantly suppressed by AMP5A in CL075-stimulated, PMA-differentiated THP-1 cultures. PMA-differentiated THP-1 were exposed to saline or AMP5A and then stimulated with 5 µg/ml CL075 for 24 hours. Cytokine/chemokine release was determined by 48-multi-plex array, and data are presented as fold change ± STD or percent inhibition (*n* = 3, *p*-value = ≤ 0.05 for fold change versus theoretical fold change of 1)

Cytokine/Chemokine	Gene ID	fold change	STD	% Inhibition	*p* value
CXCL10	CXCL10	0.03	0.02	97%	0.00010
IL-6	IL6	0.08	0.09	92%	0.00287
IL-12p40	IL12B	0.09	0.01	91%	0.00004
MCP-1	CCL2	0.10	0.01	90%	0.00007
MCP-3	CCL7	0.14	0.05	86%	0.00110
IL-1β	IL1B	0.30	0.10	70%	0.00686
MIP-1β	CCL4	0.32	0.05	68%	0.00181
MIP-1α	CCL3	0.33	0.07	67%	0.00352
IL-12p70	IL12A	0.37	0.07	63%	0.00384
MIG/CXCL9	CXCL9	0.43	0.11	57%	0.01174
IL-1RA	IL1RN	0.43	0.22	57%	0.04451
TNFα	TNF	0.44	0.05	56%	0.00308
RANTES	CCL5	0.47	0.01	53%	0.00836
IL-27	IL27	0.47	0.03	53%	0.00100
GM-CSF	CSF2	0.48	0.10	52%	0.01103
G-CSF	CSF3	0.52	0.15	48%	0.02971
IL-15	IL15	0.59	0.05	41%	0.00443
Fractalkine	CX3CL1	0.65	0.12	35%	0.03597
IL-17F	IL17F	0.67	0.10	33%	0.02683
IL-18	IL18	0.67	0.07	33%	0.01688
IL-5	IL5	0.68	0.08	32%	0.02198
GROα	CXCL1	0.70	0.05	30%	0.00809
TGFα	TGFA	0.72	0.04	28%	0.00647
MDC	CCL22	0.80	0.04	20%	0.01443
IL-2	IL2	0.80	0.08	20%	0.04981
EGF	EGF	0.81	0.07	19%	0.04440

These quantified ELISA array results were then utilized to calculate biologically relevant cytokine ratios, which have been used to assess the immunological state of cells during inflammation. The ratio of IL-12 to IL-10, for example, has been established as a predictive marker of clinical course in multiple sclerosis [26] as well as disease severity in viral infections [27]. As shown in Table 3, treatment of CL075-activated, PMA-differentiated THP-1 cells with AMP5A significantly reduced the ratio of IL-12p40/IL-10 released from 10.3 ± 3.5 seen in the saline control group to 1.3 ± 0.7 (*p*-value = 0.01). Similarly, IL-6/IL-10 ratios can be used to predict the prognosis of multiorgan dysfunction syndrome and mortality after trauma [28] as well as the severity of disease in both pneumonia [29] and COVID-19 [30]. As with IL-12/IL-10, AMP5A treatment significantly reduced IL-6/IL-10 ratio of these cultures (8.0 ± 4.1 for saline versus 0.6 ± 0.2; *p*-value = 0.04). These findings suggest that AMP5A is acting to biologically modulate the function of these cells in meaningful ways, as measured by phenotypic change, rather than simply inhibiting cytokine release.

**Table 3:** Significant changes in IL-12p40 and IL-6 to IL-10 ratios induced by AMP5A. PMA-differentiated THP-1 were exposed to saline or AMP5A and then stimulated with 5 µg/ml CL075 for 24 hours. The release of IL-12p40, IL-6, and IL-10 was then determined using a 48-multi-plex array. The resulting measurements were then used to calculate the ratio of IL-12p40 or IL-6 to IL-10 in these cultures. Data are presented as Mean ratios ± STD (*n* = 3, *p*-value = ≤ 0.05)

Biologically relevant cytokine ratios	Mean	Std	*N* = 1	*N* = 2	*N* = 3
IL-12p40/IL-10 for Saline + CL075	10.3	3.5	10.0	6.9	13.9
IL-12p40/IL-10 for AMP5A + CL075	1.3	0.7	1.2	0.7	2.0
IL-6/IL-10 for Saline + CL075	8.0	4.1	8.7	3.6	11.8
IL-6/IL-10 for AMP5A + CL075	0.6	0.2	0.4	0.7	0.7

### In silico analysis of array cytokines and chemokines reveals that proteins inhibited by AMP5A are associated with hyper-inflammatory disease states, immune cell maturation, and pro-inflammatory transcription factor activity

For contextual placement of the biologic processes underlying cytokines and chemokines regulated by AMP5A, gene enrichment analysis was performed by querying Enrichr [31, 32] or Ingenuity Pathway Analysis (IPA) software using the corresponding gene symbols (Table 4).

**Table 4:**
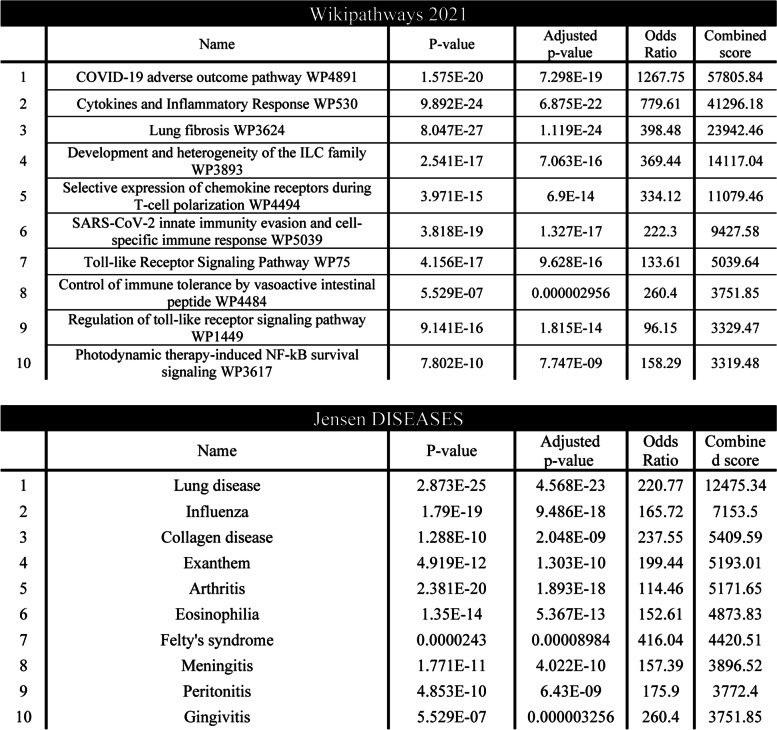
Enrichr Wikipathways and Jensens Disease Enrichment Analysis. Enrichment analysis results of differentially expressed gene set established by 48-plex cytokine/chemokine arrays in saline-treated versus AMP5A-treated PMA-differentiated THP-1 activated with 5 µg/ml CL075 for 24 hours as queried by Enrichr. Data presented as tables of significantly enriched terms in Wikipathways and Jensens Disease libraries, ranked by enrichment score

When sorted by enrichment score, the top term returned from the Wikipathways library of Enrichr was the ‘COVID-19 adverse outcome pathway’ (WP4891) followed by others such as ‘cytokine responses and inflammatory response’ (WP530) or ‘toll-like receptor signaling’ (WP75) as well as ‘lung fibrosis’ (WP3624) and ‘lymphoid development/polarization’ (WP3893 and WP4494). In addition, mining of the Jensen disease-associated databases predicts that these AMP5A-inhibited cytokines and chemokines are associated with lung disease, influenza, collagen disease, and arthritis.

**Fig. 5:**
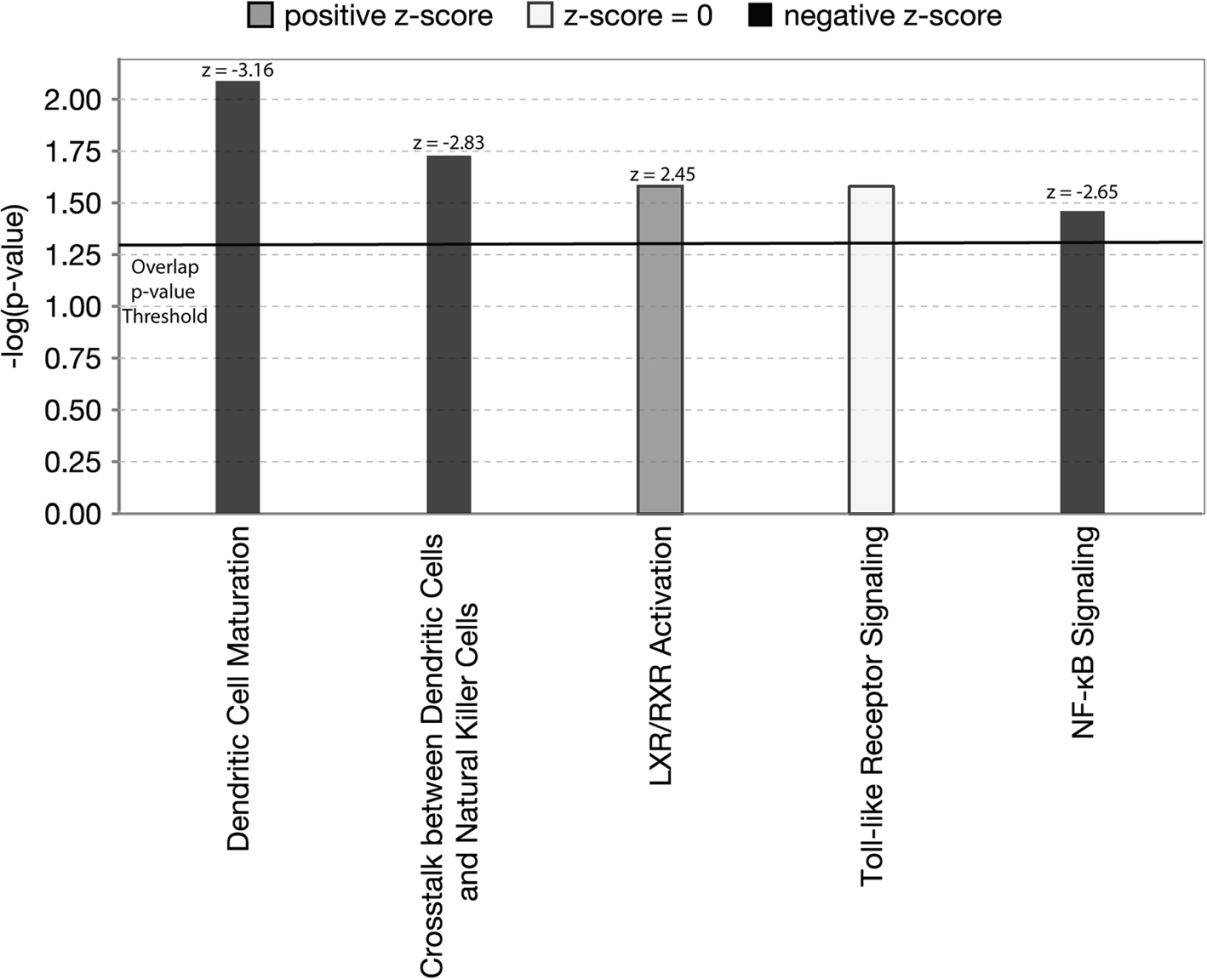
Canonical pathway analysis shows AMP5A directional regulation of cytokines and chemokines are predicted to inhibit pathways associated with increased inflammation. Log2 fold-changes and p-values of cytokines and chemokines from the 48-plex cytokine array comparing saline-treated versus AMP5A-treated PMA-differentiated THP-1 cells activated with 5 µg/ml CL075 for 24h were uploaded into IPA for canonical pathway analysis. Dark grey = pathway predicted to be activated, z-score > 2, black = pathway predicted to be inhibited, z-score < -2, white = directional regulation unable to be predicted, z = 0

Canonical pathway analysis performed in IPA showed that the cytokines and chemokines inhibited by AMP5A were predicted to significantly overlap with 6 pathways, and of these, ‘dendritic cell maturation’, ‘crosstalk between dendritic cells and natural killer cells’, and ‘NF-κB signaling’ pathways were predicted to be significantly inhibited (black bars, Fig. 5) while IPA predicted activation of ‘liver X receptor/retinoid X receptor activity’ (grey bars, Fig. 5). Overall, these data suggest that hyperinflammatory disease states, immune cell maturation, and proinflammatory transcription factor activity may be suppressed with AMP5A treatment.

## Discussion

Due to the ongoing pandemic, immune responses surrounding COVID-19 are the subject of intense investigation. What has emerged, suggests that severe infection is marked by two distinct stages: immunosuppression followed by hyperinflammation [33]. The first stage in this progression is believed to be triggered by a series of viral defenses designed to evade the immune system. Genomic studies demonstrate that coronaviruses encode for proteins that serve to delay the expression of interferons and promote viral replication [4]. As a result, unchecked viral replication potentiates tissue damage, resulting in a hyperinflammatory immunopathology driven by the release of Th1 cytokines such as TNFα, IL-1β, IL-6, and IL-12 [34]. Immune profiling of bronchial lavage fluids collected from critical COVID-19 patients presents a cellular landscape in which the accumulation of activated neutrophils and inflammatory monocytes, expressing markers such as CXCL10 and IL-1, provide a microenvironment where T-cell effector function and macrophage maturation is limited [35]. Thus, identifying prophylactic and therapeutic measures to overcome the resulting uncontrolled innate inflammation is paramount in the management of collateral lung damage and cytokine storm development in COVID-19 [36].

In this study, we investigated the effect of AMP5A on cytokine and chemokine release from macrophage-like THP-1 cells as well as PBMC activated with TLR7 and 8 agonists. TLR7/8 signaling is evolutionarily designed to detect viral ssRNA, the genetic material found in SARS-CoV-2, but evidence suggests that if left unchecked, it can lead to hyperinflammatory disease. For example, studies show that coronavirus ssRNA sequences induce nearly 2-fold higher levels of TNF-α, IL-6, and IL-12 via TLR7 and 8 than those from other viruses, triggering an inflammatory response that leads to acute lung injury and mortality in mouse models [37]. TLR7 signaling has also been found to be a powerful costimulatory signal in activated T-cells, decreasing antigen thresholds and enhancing the release of pro-inflammatory cytokines including IFNγ and TNFα [38]. In light of these observations, it appears that the immune system is predisposed to overreact to SARS-CoV-2 during periods of high viral load. In our models, AMP5A inhibits the release of TLR7/8-induced pro-inflammatory cytokines and chemokines such as IL-1β, IL-6, IL-12, TNF-α, and CXCL10. Importantly, enrichment analysis of the cytokine and chemokine profiles downregulated by AMP5A treatment in our macrophage-like model indicates that these genes are associated with lung disease, lung fibrosis, and poor outcomes in COVID-19. As a result, these findings support the idea that AMP5A lessens the severity of COVID-19’s hyperinflammatory phase.

Due to some intriguing connections with autoimmunity, it is also reasonable to suspect that TLR7/8 signaling following tissue damage could contribute to COVID-19 immunopathology. It is well established that TLR7 activation plays a prominent role in self-antigen B-cell activation and plasma cell differentiation during SLE [39]. This is more controversial for TLR8 where in some studies it may protect against TLR7 activation in SLE, however, this may be cell-type and sex-dependent [13]. Importantly, these reactions appear to be the direct result of endogenous nucleic acids being presented to TLR7/8 following the internalization of IC. For example, small nuclear ribonucleoprotein particles, a hallmark autoantibody target in SLE, promote TLR7/8 driven activation of NF-κB [40] and the uptake of DNA/RNA-associated IC formed by mixing autoantibodies with cellular debris leads to the activation of dendritic cells through TLR7 [41]. Concerning COVID-19, recent studies have shown that autoantibodies, including anti-nuclear, are highly prevalent in hospitalized patients [42]. Interestingly, CXCL10 release, identified in this investigation as a robust marker of TLR7/8 immune activation, has also been shown to be part of an amplification feedback loop driven by IFNγ and TNFα release from Th1 lymphocytes in a variety of autoimmune diseases [43]. This could be a critical observation because B-cells represent a potential source of pathogenic, TLR7/8 derived, CXCL10 following tissue damage in COVID-19 and could also be a drug target in our PBMC models.

Endogenous nucleic acids can also become readily available for TLR recognition as part of innate immune responses associated with the pathology of immune-related diseases. Neutrophils are myeloid acute phase responders to infection that once activated, undergo a unique form of programmed cell death characterized by the release of web-like extracellular chromatin and mitochondrial DNA structures known as NETs [44]. TLR signaling is a key trigger of NETs formation [45] and, in fact, NETs formation by TLR8 activation has been identified as part of a self-potentiating inflammatory loop that drives chronic inflammation in psoriasis [46]. Pathologically, excess NETs formation has been implicated with disease severity, ARDS, and thrombosis in lung viral infections, including COVID-19 [45]. Of note, TLR7/8 activation of neutrophils has also been shown to promote furin-dependent proteolytic cleavage of Fcgamma receptors on dendritic cells, monocytes, and neutrophils themselves, which results in impaired IC clearance, increased complement activation and serves as a marker of disease severity in SLE [47]. Together, these findings suggest that TLR7/8 signaling may both induce and/or exacerbate the inflammation associated with COVID-19. They also provide several unexpected implications on the therapeutic uses of AMP5A. For example, it is tempting to conclude that in the context of COVID-19, induction of furin-dependent protease activity by TLR7/8 could potentially reduce viral antigen presentation [48], promote C5a prothrombotic activity [49], and enhance viral entry into cells by exposing the TMPRSS2 cleavage site on the spike protein [50]. While not directly examined in this study, neutrophil activation and NETosis merit future investigation. Furthermore, if AMP5A globally suppresses myeloid TLR7/8 signaling, we hypothesize that blocking NET inflammation could contribute to clinical efficacy.

Having established that AMP5A inhibits the release of pro-inflammatory mediators induced by TLR7/8 activation, we used *in silico* analysis to help identify the underlying mechanisms involved with drug activity in myeloid lineages. Canonical pathway enrichment predicts that the gene sets inhibited by AMP5A treatment of PMA-differentiated THP-1 cells are associated with the inhibition of NF-κB signaling. This is consistent with previous findings that AMP5A treatment significantly inhibits NF-κB subunit DNA-binding and reporter activity in LPS-activated PBMC and TNFα-stimulated HEK cells, respectively [23]. However, a large body of evidence suggests that toll-like receptors initiate signaling cascades by using distinct sets and/or combinations of adaptor proteins. For example, TLR7 is entirely dependent on the MyD88 adaptor, whereas TLR4 also relies on TRIF [51]. Furthermore, the five NF-κB monomer proteins can be combined to form up to 15 different transcription factors, which are influenced by a string of upstream events [52]. The fact that activation of TLR2, TLR3, and TLR4 causes radically different NF-κB homo- and heterodimer binding patterns functionally emphasizes this point [52]. Moreover, crosstalk between NF-κB and other signaling pathways is essential for overall transcriptional activity, and TLR ligation can activate multiple signaling cascades simultaneously [52]. Thus, TLR-induced differences in NF-κB dynamics and other cofactors cooperating at promoter sequences will undoubtedly vary depending on immunological circumstances, necessitating the need to investigate drug activity in a variety of models. Given these considerations, our findings support the notion that AMP5A can also modulate NF-κB activity in specialized myeloid subsets and alternate TLR pathways with distinct signaling dynamics. This is significant because TLR7/8 activation of NF-κB increases cytokine production [4] and, in conjunction with inflammasome activity, NF-κB drives an axis of IL-1β, IL-6, IL-12, TNFα, and Th1 T-cell hyperinflammation as part of COVID-19 immunopathology [34].

We were also able to confirm that the activation of recognized immunomodulatory transcription factors is critical for AMP5A to inhibit TLR7/8 signaling in this study. Previously, we have demonstrated that antagonism of AhR and PPARγ blocks the ability of AMP5A to inhibit TNFα and IL-1β release from PBMC [23] as well as IL-6 release from PMA-differentiated THP-1 cells activated through TLR4 using LPS [19]. It is important to remember, however, that these pathways rely heavily on the expression of the receptors themselves, interactions with other signaling pathways, and non-genomic interactions with accessory molecules such as cytoplasmic kinases, all of which may vary depending on the immune cell type [53–55]. Consequently, we wanted to ensure that other immune cell subsets correctly expressed the molecular framework required for AMP5A to suppress TLR signaling pathways originating from endosomal compartments. Using the dose-dependent nature of AMP5A-induced CL075-induced CXCL10 release from PMA-differentiated THP-1 cells, we were able to demonstrate that activation of AhR and PPARγ once again contributes to drug activity. This finding is supported by enrichment analysis, which predicts that differentially expressed gene sets derived from this model are associated with the activation of liver X and retinoid X receptors, which are known binding partners and coactivators of PPARγ [56].

A substantial body of evidence suggests that AhR and PPARγ are effective transcriptional regulators and immunomodulators. AhR has been shown to suppress NF-κB activity by increasing competitive inhibition of pro-inflammatory p50/p65 NF-κB heterodimers with anti-inflammatory p50/p50 homodimers [57] and preventing NF-κB translocation into the nucleus [58]. Similarly, PPAR can inhibit NF-κB activity by directly binding to DNA, NF-κB subunits, or coactivators, as well as promoting the production of NF-κB inhibitory proteins [56]. More importantly, activation of these transcription factors has been shown to restrict the release of cytokines from a variety of cells, modulate the epigenetic status of regulatory immune cell subsets, and protect against endotoxin-induced ARDS [53, 56, 59].

The impact of activating these pathways appears to go beyond simple inhibition of cytokine production in our hands. To illustrate, canonical pathway enrichment analysis revealed that dendritic cell maturation had a negative z-score, implying that the presence of AMP5A influences phenotypic patterns driven by TLR activation in THP-1 cells. This could be related to the change in the expression ratios of IL-12 or IL-L6 and IL-10 observed in these cultures as well. The progression of the inflammatory response may be influenced by these disparities in cellular differentiation. Changes in myeloid cell type, such as transitions from classical M1 macrophages to alternatively activated M2 macrophages, are recognized as hallmarks of the immune system’s passing through distinct phases leading to resolution [60]. However, research has shown that, while dendritic cells are highly regarded antigen-presenting cells, they can also limit T-cell responses when dysregulated [61, 62]. Ultimately, skewing transcriptional programming patterns *in vivo* could help the immune system navigate immune checkpoints more effectively and/or potentially counteract the T-cell exhaustion seen in severe COVID-19 patients

This investigation does have limitations. To begin, the cytokine arrays used to generate the gene sets for enrichment analysis may be biased toward pro-inflammatory transcription factors such as NF-κB or inflammatory disease. Second, focusing on CL075-induced CXCL10 release from PMA-differentiated THP-1 cells may not accurately reflect how AMP5A regulates other cytokines and chemokines. Finally, the metabolomics and half-life of AMP5A in these cultures have yet to be determined, but they may play a role in determining the overall temporal implications of treatment. We believe, however, that the findings presented in this report provide solid evidence that AMP5A inhibits pro-inflammatory myeloid signaling via TLR7 and 8 by activating the immunomodulatory transcription factors AhR and PPARγ.

## Conclusion

Due to the biphasic course of COVID-19, different therapeutic strategies may be more appropriate for the 'immunosuppressive' as opposed to the 'hyperinflammatory' phases. Early intervention should focus on enhancing antiviral immunity, while later strategies should focus on suppressing inflammation. In this report, we present evidence that AMP5A can suppress TLR 7 and/or 8 signaling in a variety of myeloid cells, as well as the rationale for targeting this pathway during the hyperinflammatory stage of COVID-19 and autoimmune diseases such as SLE. AMP5A treatment of innate immune cells results in a significant decrease in CXCL10 release as well as inhibition of a slew of pro-inflammatory mediators linked to hyperinflammation and lung disease. Furthermore, data show that AMP5A-induced suppression of CXCL10 is directly related to the activation of immunomodulatory AhR and PPAR signaling pathways. These findings suggest that the modes and mechanisms of action of AMP5A are well suited to address the hyperinflammation seen in severe COVID-19 patients, as well as conditions with dysregulated TLR activation, such as autoimmunity.

## Data Availability

The data analyzed in this report are included in published articles or available from the corresponding author on reasonable request.

## Abbreviations

TNFα: Tumor necrosis factor-alpha
IFNγ: Interferon gamma
IL-1β: Interleukin 1 beta
IL-2: Interleukin 2
IL-6: Interleukin 6
IL-12: Interleukin 12
CXCL9: C-X-C motif chemokine ligand 9
CXCL10: C-X-C motif chemokine ligand 10
CXCL11: C-X-C Motif chemokine ligand 11
M-CSF: Macrophage colony-stimulating factor
GM-CSF: Granulocyte-macrophage colony-stimulating factor
MCP: Monocyte chemoattractant protein
MIP: Macrophage inflammatory protein
RANTES: Regulated upon Activation, Normal T-cell Expressed, and Secreted or CCL5
COVID-19: Coronavirus disease of 2019
SARS-CoV-2: Severe acute respiratory syndrome coronavirus 2
Th1: CD4+ T helper 1 T cell
TLR: Toll-like receptor
TLRs: Toll-like receptors
TRIF: TIR-domain-containing adaptor-inducing interferon-β
NETs: Neutrophil extracellular trap
ARDS: Acute respiratory distress syndrome
SLE: Systemic lupus erythematosus
IC: Immune complex
AMP5A: Low molecular weight fraction of 5% human serum albumin
NF-κB: Nuclear factor kappa-light-chain-enhancer of activated B cells
p50: NF-κB subunit p50
p65: NF-κB subunit p65 or RelA
RelB: NF-κB subunit RelB
LPS: Lipopolysaccharide
PBMC: Peripheral blood mononuclear cells
PMA: Phorbol 12-myristate 13-acetate
IQR: Interquartile Range
AhR: Aryl hydrocarbon receptor
PPAR: Peroxisome proliferator-activated receptor
HEK: Human embryonic kidney
THP-1: Human acute monocytic leukemia cells
REP: Relative potency
ssRNA: Single-stranded ribonucleic acid
TMPRSS2: Type 2 transmembrane serine protease
C5a: Complement 5a
